# On the Retrogression of Labor

**Published:** 1859-09

**Authors:** 


*On the Retrogression of Labor.* By Dr. CHARTEN.

C. directs attention to the fact, that at any period of pregnancy, but particularly during the later months, labor may commence and proceed regularly, so far as that the os uteri is opened up so considerably as to put the projecting bag of membranes on the stretch, and yet not only does complete rest recur, but the retrogression is so complete, that the os uteri closes again till some weeks after, when the labor begins afresh, and proceeds uninterruptedlly to its close. In evidence of this, he cites four cases:—The first occurred in the Obstetrical Clinique at Paris. A woman, eight months pregnant, was seized with regular pains; the os uteri opened to the size of a five france piece and was soft; towards evening the water came away; during the night the pains continued regular, but at 4 A. M. ceased entirely; the os uteri gradully closed, and, by the following evening, was quite shut. Twenty-one days after, the labor set in anew, and proceeded regularly to its termination. In the second case, the birth took place 32 days; in the third, 35 days; and in the fourth, 22 days after the first occurrence of labor.

*Ed. Med. Journal.*

